# Robot-assisted sacrocolpopexy: not only for vaginal vault suspension? An observational cohort study

**DOI:** 10.1007/s00192-021-04740-y

**Published:** 2021-06-23

**Authors:** Femke van Zanten, Egbert Lenters, Ivo A. M. J. Broeders, Steven E. Schraffordt Koops

**Affiliations:** 1 Department of Gynecology, Meander Medical Center, Maatweg 3, 3813 TZ Amersfoort, The Netherlands; 2 Faculty of Science and Technology, Institute of Technical Medicine, Twente University, Enschede, The Netherlands; 3 Department of Surgery, Meander Medical Center, Amersfoort, The Netherlands

**Keywords:** Female pelvic organ prolapse, Recurrence, Robot-assisted surgery, Sacrocolpopexy, Sacrocervicopexy

## Abstract

**Introduction and hypothesis:**

Surgery for pelvic organ prolapse (POP) has high recurrence rates. Long-term anatomical and patient-reported outcomes after pelvic floor repair are therefore required.

**Methods:**

This prospective observational cohort study was conducted in a teaching hospital with tertiary referral function for patients with POP. Patients with symptomatic vaginal vault or uterine prolapse (simplified POP Quantification [sPOPQ] stage ≥2), who underwent robot-assisted sacrocolpopexy (RASC) or supracervical hysterectomy with sacrocervicopexy (RSHS), were included. Follow-up visits with sPOPQ evaluations were planned 4 years after surgery. Patients received pre- and postoperative questionnaires reporting symptoms of vaginal bulge, Urogenital Distress Inventory (UDI-6), and Pelvic Floor Impact Questionnaire (PFIQ-7). Primary outcome was patient self-reported symptoms. Secondary outcome was anatomical cure (sPOPQ stage 1) for all vaginal compartments.

**Results:**

Seventy-seven patients were included. Sixty-one patients (79%) were evaluated after 50 months (physical examination *n* = 51). Symptoms of bulge (95% vs 15% *p* ˂ 0.0005), median UDI-6 scores (26.7 vs 22.2, *p* = 0.048), median PFIQ-7 scores (60.0 vs 0, *p* = 0.008), and median sPOPQ stages in all landmarks improved significantly from the pre- to the postoperative visit. Thirty patients (59%) were completely recurrence free and 96% of patients had no apical recurrence. Most recurrences were asymptomatic cystoceles (20%). There was one surgical re-intervention for recurrent prolapse (1.6%).

**Conclusions:**

Robot-assisted sacrocolpopexy and RSHS show sustainable results in the treatment of prolapse. Symptoms of bulge, urinary symptoms, and quality of life improved substantially 50 months postoperatively. Patients should be counseled about the risk of anterior wall recurrence and the small chance of recurrent symptoms that need treatment.

## Introduction

About 1 in 6 women (11–19%) undergo a surgical pelvic organ prolapse (POP) correction due to prolapse or urinary incontinence-related complaints [1]. High recurrence rates are found after surgical repair of female POP [2, 3]. Vaginal vault prolapse is common and specifically recurrences in the anterior compartment are a recognized long-standing problem [2–4]. Determination of long-term outcomes for the patient after prolapse surgery is therefore essential. Open abdominal sacrocolpopexy (ASC) has been shown to result in a lower recurrence of vault prolapse than the vaginal approach to prolapse, but is associated with a longer return to daily activities [2]. In order to avoid this long recovery time, a minimally invasive approach to sacrocolpopexy has been used. The current literature describes objective cure rates for the apical compartment to be 97–100% after robot-assisted sacrocolpopexy (RASC) [5]. However, these results are mostly based on short- to mid-term time frames. Only a few studies describe outcomes more than 24 months after surgery [5, 6]. Long-term postoperative results on patient-reported outcomes are lacking. This study was set up because of this knowledge gap.

Female POP influences quality of life (QoL) as well as day-to-day activities, emphasizing the need for long-term subjective results even more [7]. We evaluated whether RASC or robot-assisted supracervical hysterectomy with sacrocervicopexy (RSHS) leads to both long-term improved subjective patient-reported outcomes as well as anatomical results.

## Materials and methods

**Fig. 1:**
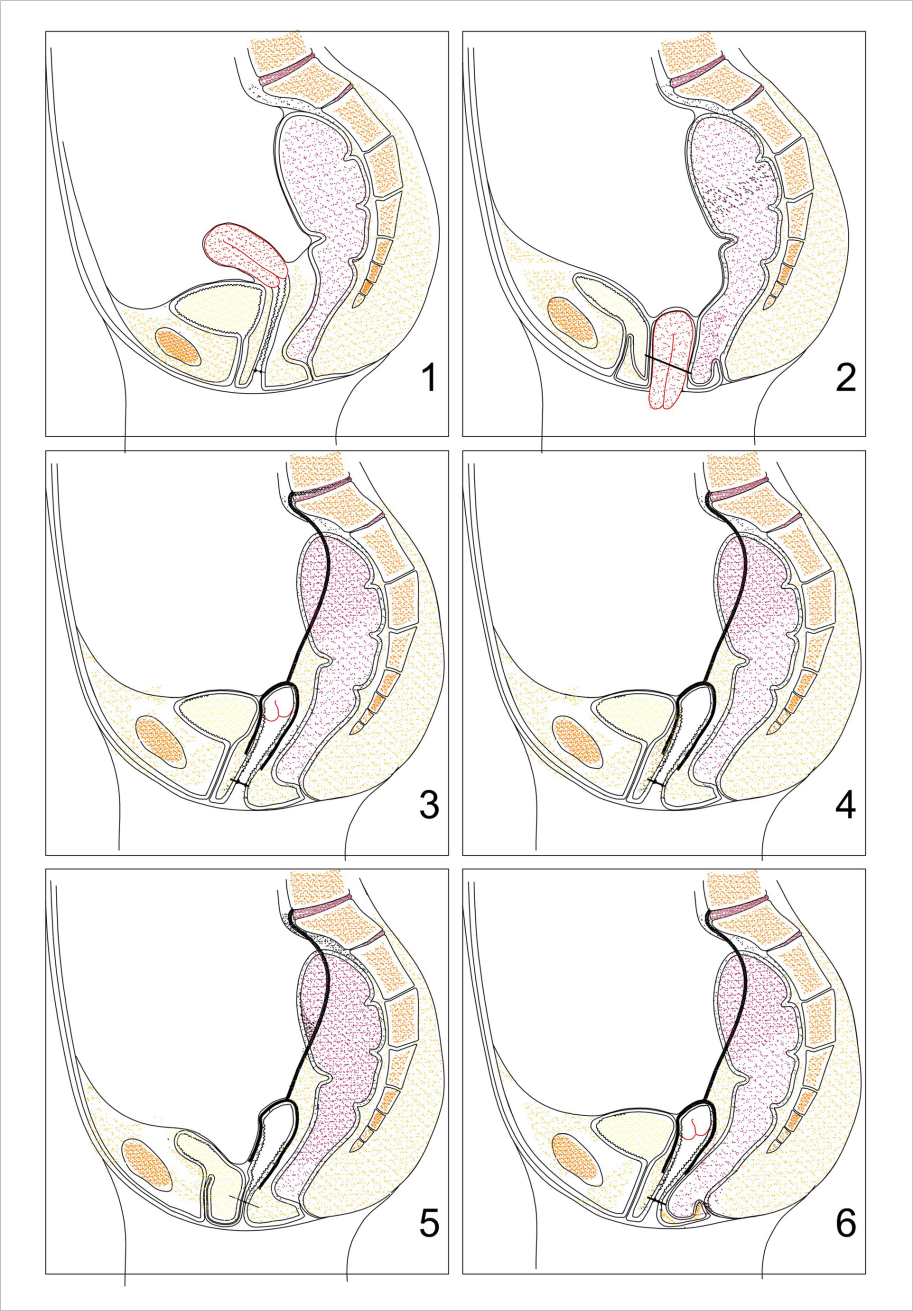
Example of prolapse before and after surgery and optimal surgical outcomes. Simplified Pelvic Organ Prolapse Quantification (sPOPQ) stage 1 describes either no prolapse or a minimal prolapse (>1 cm above the hymnal remnants). In stage 2, the given point descends 1 cm above and 1 cm below the hymnal remnants. Stage 3 describes a prolapse that descends more than 1 cm beyond the hymenal remnants, but does not represent stage 4, which includes complete vaginal vault eversion or complete procidentia uteri. Stage 0 does not exist by definition of the sPOPQ system. *1* No prolapse. *2* Stage 3 prolapse of bladder, uterus, and rectum. *3* deal anatomical situation after robot-assisted supracervical hysterectomy with sacrocervicopexy (RSHS). *4* Ideal anatomical situation after robot-assisted sacrocolpopexy (RASC). *5* Stage 3 prolapse of anterior wall after RASC. *6* Stage 2 prolapse of posterior wall after RSHS. The black line represents the hymnal remnants

All patients with symptomatic vaginal vault prolapse or uterine prolapse, who underwent RASC or RSHS in 2011 and 2012, were included. Stages of prolapse were identified with the aid of simplified Pelvic Organ Prolapse Quantification (sPOPQ) [8]. sPOPQ describes four vaginal landmarks (Ba: anterior vaginal wall; Bp: posterior vaginal wall; C: vaginal cuff/cervix; D: fornix posterior). Examples of the sPOPQ stages are shown in Fig. 1. Patients were treated in our hospital with tertiary referral function for patients with pelvic organ prolapse (POP). Surgery was performed by two urogynecologists. Patients were advised of alternative treatments available to them and informed about the risks and benefits of the procedure. Inclusion criteria were patients with symptomatic vaginal vault prolapse or descensus uteri sPOPQ stage ≥2. Exclusion criteria were a poor health status with an inability to undergo general anesthesia, age ˂ 18 years, ≥ 3 laparotomic surgeries, planned pregnancy, and known pelvic malignancies. This study was judged to be exempt by the National Central Committee on Research Involving Human subjects (CCMO) as it was an observational cohort study. Follow-up visits with questionnaires were part of the routine follow-up of patients with mesh implants.

Primary outcome measurements were patient-reported outcomes on QoL and pelvic floor functions. Examination of patients and evaluation of complaints with the questionnaire were obtained preoperatively and at 1 and 4 years postoperatively. The questionnaires included questions regarding symptoms of vaginal bulge (seeing and/or sensation), micturition symptoms (Urogenital Distress Inventory; UDI-6) [9], and QoL (Pelvic Floor Impact Questionnaire; PFIQ-7) [10]. The PFIQ-7 includes the Incontinence Impact Questionnaire (IIQ-7), Colorectal-Anal Impact Questionnaire (CRAIQ-7), and Pelvic Organ Prolapse Impact Questionnaire (POPIQ-7) [11]. Participants answered the PFIQ-7 using one of four options: “not at all (0),” “slightly (1),” “moderately (2),” “greatly (3)”). Each subscale ranges from 0 to 100 (mean score × 33 1/3). The total score is the sum of all three subscales (0–300). A higher score indicates an increased negative impact on daily life.

Secondary outcome measure was objective anatomical cure rate, defined as sPOPQ stage 1 for all anatomical landmarks. Definition of recurrence was as follows: sPOPQ stage ≥2 in any of the compartments. Retreatments regarding recurrent prolapse were scored. Patients with no postoperative consultation available, and who also did not send in a questionnaire, were considered lost to follow-up.

### Surgical technique

The surgical technique used has been described previously [11]. In short, all procedures were performed with robotic assistance using the da Vinci Si HD (Intuitive Surgical, Sunnyvale, CA, USA). Prolene mesh was used (Prolene, Ethicon, Johnson & Johnson, Hamburg, Germany). Attachment to the sacral promontory was performed using titanium tacks (Autosuture Protack 5 mm; Covidien, Mansfield, MA, USA). Distally, the mesh was attached using non-absorbable sutures (Ethibond; Ethicon) to the anterior and posterior vaginal wall and to the vaginal apex/cervix. Two meshes were used, configured into a “Y” shape intracorporeally. If the uterus was present, a supracervical hysterectomy was performed. No total hysterectomy was performed to diminish the risk of mesh exposure. The peritoneum was closed using a 23-cm V-Loc suture (Covidien). At the end of the procedure, a vaginal examination was performed by the urogynecological surgeon to evaluate the correction of the prolapse. Postoperatively, all of the patients were prescribed a laxative (Macrogol 3350/electrolytes, Movicolon; Norgine, Hengoed, UK). Patients were advised to refrain from postoperative heavy lifting and sexual intercourse for 6 weeks postoperatively.

### Statistical analysis

Statistical analysis was performed using SPSS v. 22.0 (IBM, Armonk, NY, USA). A *p* value of <0.05 was considered significant. Data were presented as mean ± SD or median and range for normally and non-normally distributed continuous values respectively. Number and percentages were used for nominal and categorical values. Independent samples t test, Mann–Whitney *U* test, and Chi-squared test were used to compare data for mean, median, and nominal values respectively. Paired t test, Wilcoxon signed rank test, and McNemar’s test were used to compare scores before and after surgery as appropriate.

## Results

**Fig. 2:**
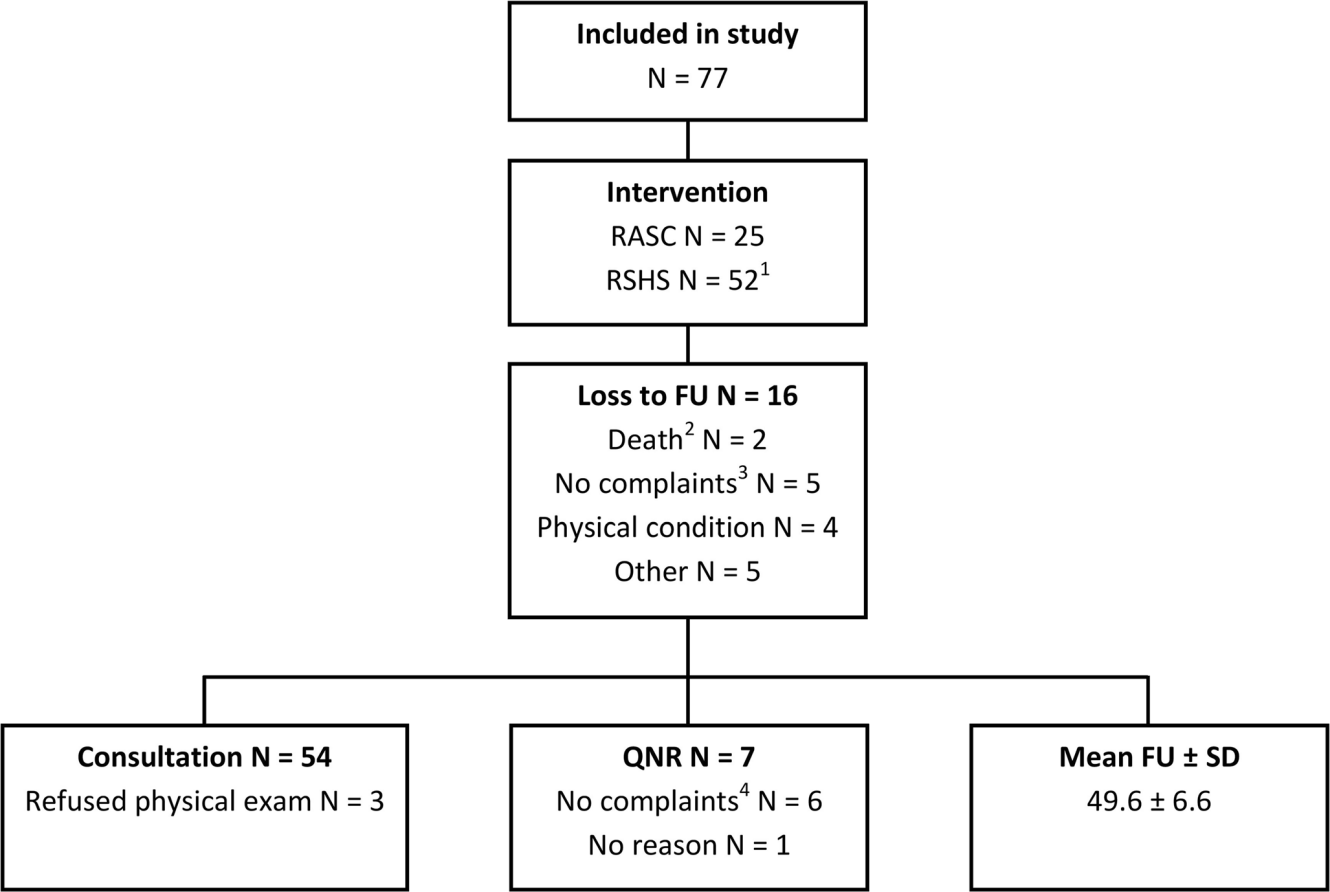
Flow chart of included patients. ^1^One hysteropexy, decided during surgery for technical reasons. ^2^Owing to natural causes. ^3^Patients had no complaints and therefore refused consultation. ^4^Patients had no complaints and therefore declined consultation in the outpatient clinic, but did return a questionnaire. FU follow-up, QNR questionnaire, SD standard deviation

In total, 77 patients were included (Fig. 2). Patients had a mean age of 63.1 ± 10.3 years and BMI of 26.0 ± 3.5 (Table 1). One surgery was converted to an open procedure owing to anesthetic-related problems. Ten patients (13%) had a concomitant placement of a transobturator tension-free vaginal tape (TVT-O) because of severe preoperative stress urinary incontinence (SUI). A concomitant anterior (AC) or posterior colporrhaphy (PC) was performed in 11 patients (14.3%; RASC *n* = 2; RSHS *n* = 9). In 61 patients (79.2%) long-term follow-up was available, with a mean follow-up of 49.6 ± 6.6 months. Of these 61 patients, 7 responded with a questionnaire only: the questionnaire was sent back by mail. Patients who were seen for follow-up in our outpatient clinic were examined by an independent (not the surgeon) researcher (FZ). During consultation, 3 patients declined physical examination, because these patients judged this examination unnecessary as they had no complaints.

**Table 1:** Baseline demographics *N* = 77

Demographic	Data
Age	63.1 ± 10.3
BMI	26.0 ± 3.5
Parity	3 (0–11)
Postmenopausal	63 (81.8)
Previous hysterectomy	25 (32.5)
Previous POP/incontinence surgery	29 (37.7)
History of intra-abdominal surgery^a^	33 (42.9)
Sphincter rupture labor	2 (2.6)
Episiotomy labor	35 (45.5)
COPD	6
ASA score	
1	25 (32.5)
2	49 (63.6)
3	3 (3.9)
Sexually active	
No	27 (35.1)
Yes	40 (51.9)
Not reported	10 (13.0)
Smoking (active)	14 (18.2)^c^
Vaginal estrogen use	2 (2.6)
Preoperative sPOPQ	
Ba	2.3 ± 1.0
Bp	2.2 ± 1.0
C	2.2 ± 1.0
D^b^	1.4 ± 0.9
Pre-operative SUI	35 (45.4)
Pre-operative UUI	32 (41.6)

Numbers are presented as mean ± SD, median (range) or number (%)

*ASA* American Society of Anesthesiologist, *BMI* body mass index, *POP* pelvic organ prolapse, *sPOPQ* simplified pelvic organ prolapse quantification, *SUI* stress urinary incontinence, *UUI* urge urinary incontinence

^a^Includes no POP surgery

^b^Only in patients with uterus in situ

^c^Eleven unknown

### Patient-reported outcome measures

Symptoms of bulge improved from 95% to 10% (*p* < .0005; Table 2). Quality of live scores also improved significantly, mainly because of improved urinary and POP impact scores. Colorectal QoL scores were low both pre- and postoperatively, and did not change. The total UDI-6 scores after 4 years improved significantly (*p* = 0.048). Exploring the three subdomains, improvement of urinary symptoms was mostly caused by enhancement of obstructive micturition. Three patients (4.9%) needed a TVT-O postoperatively. Of the 10 patients receiving a TVT during surgery, 2 had persistent complaints of moderate SUI.

**Table 2:** Patient-reported outcome measures

	Preoperative *N* = 77	Postoperative *n* = 61	*p* value
Bulge symptoms	73 (94.8)	6/61 (9.8)	˂0.0005
PFIQ-7 total (0–300)	60.0 (0–185.7)	0 (0–300)	0.008
UIQ-7 (0–100)	16.7 (0–90.5)	0 (0–100)	0.016^*^
CRAIQ-7 (0–100)	0 (0–57.1)	0 (0–100)	0.051
POPIQ-7 (0–100)	31.0 (0–95.2)	0 (0–100)	0.005^*^
UDI-6 total (0–100)	26.7 (0–93.3)	22.2 (0–72.2)	0.048^*^
Irritative (0–100)	33.3 (0–100)	33.3 (0–100)	0.450
Stress (0–100)	33.3 (0–100)	16.7 (0–100)	0.574
Obstructive (0–100)	33.3 (0–100)	0.0 (0–100)	0.008^*^

Data presented as number (percentage), median (range)

*CRAIQ-7* Colorectal-Anal Impact Questionnaire, *PFIQ-7* Pelvic Floor Impact Questionnaire, *POPIQ-7* Pelvic Organ Prolapse Impact Questionnaire, *UDI-6* Urinary Distress Inventory, *UIQ-7* Urinary Impact Questionnaire

* statistically significant

### Anatomical results

**Fig. 3:**
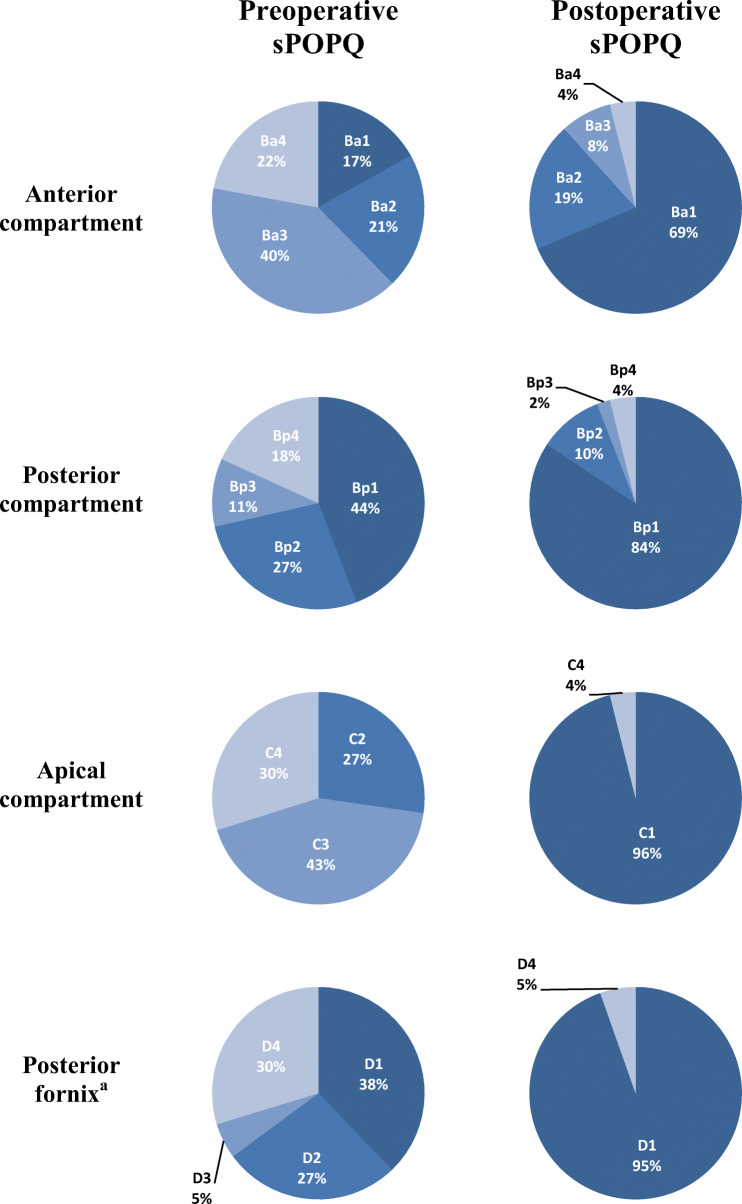
Pre- and postoperative anatomical results. Anatomical landmarks: Ba—anterior compartment, Bp—posterior compartment, C—apical compartment, D—posterior fornix. sPOPQ stages: stage 1—no prolapse, stage 2—vaginal prolapse between 1 cm above the hymen and 1 cm below the hymen, stage 3—vaginal prolapse >1 cm below the hymen, but not totally everted, stage 4—total vaginal eversion. *sPOPQ* simplified pelvic organ prolapse quantification. ^a^Only when the cervix is present

The pre- and postoperative stages of the sPOPQ for all patients seen in the outpatient clinic for follow-up are shown in Fig. 3. Thirty patients (30/51; 58.8%) were completely recurrence free in all compartments at final follow-up. With usage of a strict definition of recurrence as sPOPQ stage 2 or higher, the highest recurrence rate was found in the anterior compartment. Most patients presenting with postoperative POP were shown to have a mild cystocele grade 2 with no symptoms of vaginal bulge (19.6%). After 50 months two stage 4 recurrent apical prolapses were detected (3.9%). These 2 patients had a stage 4 apical prolapse preoperatively. Recurrent cystocele significantly occurred more often after RSHS than after RASC (*p* = 0.022). In 61 patients it was known if a prolapse-related re-intervention was necessary. One patient underwent a surgical re-intervention (AC, 1.6%) and 1 patient received a ring pessary (1.6%).

The median sPOPQ stages of all four anatomical landmarks improved significantly from the pre- to the postoperative visit (median preoperative and postoperative stages: sPOPQ Ba: 3.0 to 1.0 [*p*˂0.0005] sPOPQ Bp: 2.0 to 1.0 [*p*˂0.0005]; sPOPQ C: 3.0 to 1.0 [*p*˂0.0005]; sPOPQ D: 2.0 to 1.0 [*p*˂0.010]).

## Discussion

Ninety-six percent of all patients had no apical recurrence at follow-up 50 months postoperatively. Thirty patients (58.8%) showed no vaginal prolapse in any compartment. Re-operation due to recurrent prolapse was needed in 1 patient (1.6%; anterior colporrhaphy) and 1 patient needed a pessary.

Only a few studies report on long-term results after sacrocolpopexy. A retrospective study on 70 patients undergoing RASC (follow-up 72 months) identified 4 patients (5.7%) who needed repeat surgery for recurrent prolapse (including 1 with apical recurrence) [12]. These numbers are in line with our outcomes. Pacquée et al. [13] performed a prospective cohort study of 331 patients who underwent laparoscopic sacrocolpopexy (LSC), with 270 patients reviewed for follow-up (*n* = 185 physical examination/interview; *n* = 95 interview). After 85.5 months, 83% reported improvement based on the Patient Global Impression of Change score. Apical recurrence was reported in 9% of patients, anterior and posterior prolapse recurrences in 22% and 29% respectively. The reintervention rate for prolapse was 3.3%, comparable with our results. The excellent report by Culligan et al. [6], on 253 patients with a 5-year follow-up, showed a 89% overall success rate and no apical recurrence. This is a higher success rate than we found. The reoperation rate in this study of 4% is low and comparable with our surgical re-intervention rate of 1.6%.

Most recurrences in our study were sPOPQ stage 2 cystoceles with no symptoms of vaginal bulge. Even though a high recurrence rate was detected for the anterior compartment, there was a substantial improvement in functional well-being, symptoms of bulge, and QoL.

The clinical relevance of these asymptomatic recurrences remains unclear. Mild prolapses on examination are often seen and they are mostly asymptomatic. Our findings are consistent with the population-based study of Slieker-ten Hove et al. [14]. This study found a high rate of stage 2 prolapse in the normal female population without any symptoms. One could argue whether the strict definition used for recurrent prolapse (≥ stage 2), like ours in this present study, is correct, as patients do not notice any symptoms. The often-used definition for prolapse, i.e., prolapse up to and beyond the hymen, might be more precise, as with this definition more patients have symptoms.

Myers et al. [15] showed that women who underwent subtotal hysterectomy versus a total hysterectomy during RASC were more likely to have a recurrent prolapse (stage ≥2 of any compartment) after 1 year. No statistically bothersome symptoms were detected. We also found a higher rate of recurrent prolapse in the subtotal hysterectomy group (*p* = 0.022). The theoretical basis of this difference might be a difference in the dissection of the anterior wall and the tensioning of the mesh on the anterior part. The OPTIMAL trial also shows the challenge of prolapse surgery in repairing all compartments [16]. This randomized trial compared vaginal prolapse surgery. Uterosacral ligament suspension was compared with sacrospinous ligament fixation and showed an estimated surgical failure rate of 61.5% and 70.3% respectively. One can conclude that treatment of the anterior compartment remains challenging, with all the different methods described [4, 17].

Loss of apical support often occurs in women with anterior wall prolapse that extends beyond the hymen [18]. After combined apical and cystocele repair procedures, a significantly lower prolapse reoperation rate was seen than in women with an isolated anterior wall repair (11.6 vs 20.2%, *p* < .01) [18]. In this study, 83% of women had a cystocele preoperatively (22% stage 4), compared with 31% postoperatively (4% stage 4). As opening the vagina during AC could theoretically lead to more mesh exposure, further research should first be performed, before combining RASC with concomitant AC.

The degree of anterior dissection and tensioning of the mesh are important steps in RASC, but mostly based on experience [19]. Recent studies suggest that more caudal anterior dissection could lead to fewer recurrences. However, mesh placement that is to far caudally may possibly lead to new functional symptoms such as urine incontinence [19, 20]. Future research should be focused on this subject.

Studies describing QoL after RASC based on validated questionnaires are scarce. One large cohort study (*N* = 150) with 1-year follow-up evaluations, showed improved PFIQ-7 scores from 59 to 6.5 (*p*˂.0001) [21], which is in line with our scores.

Strengths of this study are the long follow-up period and the prospective design. Loss to follow-up was known in most cases. Some of these patients were willing to return validated questionnaires via mail, improving our ability to measure long-term results. Limitations are the use of a single tertiary referral center and the small sample size. This limited generalizability and the performance of a logistic regression analysis. Another limitation is the heterogeneity of this cohort, as for the middle compartment prolapse, both women vaginal vault prolapse and women with hysterocele were included.

With the rising incidence of female POP and treatments for POP in our aging population, long-term results are increasingly relevant. RASC and RSHS show sustainable results in the treatment of prolapse. After a follow-up of 50 months 96% of patients showed no apical recurrence. Patients should be counseled preoperatively about the risk of a recurrent anterior wall prolapse, for which a small percentage needs treatment.
